# Centriole movements in mammalian epithelial cells during cytokinesis

**DOI:** 10.1186/1471-2121-11-34

**Published:** 2010-05-21

**Authors:** Asta Björk Jonsdottir, Roeland W Dirks, Johannes Vrolijk, Helga M Ögmundsdottir, Hans J Tanke, Jorunn E Eyfjörd, Karoly Szuhai

**Affiliations:** 1 Cancer Research Laboratory, Faculty of Medicine, University of Iceland, Vatnsmyrarvegi 16, 101 Reykjavik, Iceland; 2 Department of Molecular Cell Biology, Leiden University Medical Center, Einthovenweg 20, 2300 RC Leiden, the Netherlands

## Abstract

**Background:**

In cytokinesis, when the cleavage furrow has been formed, the two centrioles in each daughter cell separate. It has been suggested that the centrioles facilitate and regulate cytokinesis to some extent. It has been postulated that termination of cytokinesis (abscission) depends on the migration of a centriole to the intercellular bridge and then back to the cell center. To investigate the involvement of centrioles in cytokinesis, we monitored the movements of centrioles in three mammalian epithelial cell lines, HeLa, MCF 10A, and the p53-deficient mouse mammary tumor cell line KP-7.7, by time-lapse imaging. Centrin1-EGFP and α-Tubulin-mCherry were co-expressed in the cells to visualize respectively the centrioles and microtubules.

**Results:**

Here we report that separated centrioles that migrate from the cell pole are very mobile during cytokinesis and their movements can be characterized as 1) along the nuclear envelope, 2) irregular, and 3) along microtubules forming the spindle axis. Centriole movement towards the intercellular bridge was only seen occasionally and was highly cell-line dependent.

**Conclusions:**

These findings show that centrioles are highly mobile during cytokinesis and suggest that the repositioning of a centriole to the intercellular bridge is not essential for controlling abscission. We suggest that centriole movements are microtubule dependent and that abscission is more dependent on other mechanisms than positioning of centrioles.

## Background

A centrosome consists of a pair of centrioles surrounded by pericentriolar material, and it duplicates once during the cell cycle. The two centrioles have different structures and function. The older "mother" centriole is associated with centriolar appendages, specific proteins such as cenexin and centrobin, it attaches microtubules and supports ciliogenesis. The younger "daughter" centriole lacks all these structures [1-4]. The centrosome duplication begins in G_1 _by separation of the centrioles. At early S phase procentrioles start to nucleate near the base of the pre-existing centrioles, that then elongate and mature [5,6]. As a cell exits G_2 _each centrosome nucleates microtubules and the mitotic spindle is formed [7]. Although cells with depleted centrosomes can divide, the presence of centrosomes ensures efficient formation of the mitotic spindle and facilitates cell division [8-10]. Cells with supernumerous centrosomes can form multipolar spindles leading to serious aberrations in chromosomal segregation [11,12]. Cytokinesis starts during anaphase, when the microtubules gradually concentrate at the spindle midzone and a perpendicular ring of actomyosin contracts to form a cleavage furrow. Subsequently, the cleavage furrow ingresses and an intercellular bridge is formed containing the midbody [13,14]. The midbody consists of overlapping microtubules and additional proteins, many of which are required for cytokinesis. These proteins are mainly secretory and membrane-trafficking proteins, actin and microtubule associated proteins and protein kinases [15,16]. Contractile ring assembly is directed by the RhoA guanosine triphosphatase (GTPase) and the non-muscle myosin II is among proteins required for furrow formation [17-19]. Cytokinesis is terminated by midbody cleavage (abscission) [20,21] and each daughter cell then receives only one centrosome [22]. The process of abscission is carefully regulated [16,23,24], but the control mechanisms have not been fully elucidated. Many studies support the idea that the centrosomes facilitate the final division into two daughter cells. It has been postulated that they promote and regulate to some extent the abscission phase of cytokinesis by acting as a scaffold or by providing essential signaling molecules [9,22]. Also, a role for the centrioles has been suggested in determining abscission. Reports of possible centriole movements prior to abscission go back to 1973 when the centrioles were studied by using electron microscopy. It was then noticed that in late cytokinesis centrioles were present at a region near the midbody [25], and these observations were supported by later publications [26,27]. Studies have shown that when the cleavage furrow has been formed one of the two centrioles moved to the intercellular bridge, and then back to the cell center. This type of movement was observed in 90% of analyzed HeLa cells [28]. Centriolin, a maternal component of centrioles, localizes also to the intercellular bridge and it has been shown that silencing centriolin causes cells to have severe difficulties in completing cytokinesis and that the dividing cells remain interconnected [3,29]. Centriolar repositioning to the midbody is therefore thought to be important for the termination of cytokinesis.

In a previous study we observed that cytokinesis was delayed in *BRCA2 *heterozygous primary cells [30], which prompted us to investigate the process of cytokinesis in more detail. We asked whether the migration of a centriole to the intracellular bridge is part of a general mechanism and required for completion of cytokinesis. To this end we analyzed the movements of fluorescently labeled centrioles during cytokinesis by time-lapse imaging in three different mammalian epithelial cell lines. Our observations indicate that the suggested key function of centrioles at the intercellular bridge in controlling the abscission phase of cytokinesis [28] may not be universally valid and could be cell-line dependent.

## Results

### Characterization and quantitative analysis of centriole mobility during cytokinesis

To investigate whether centrioles play a key role in regulating abscission and thereby in completing cytokinesis we analyzed their positioning and movements in human as well as in mouse epithelial cells by time-lapse fluorescence imaging using centrin1-EGFP as a marker.

Centrin1-EGFP and α-Tubulin-mCherry were co-expressed in both human mammary epithelial MCF 10A and HeLa cells by transient transfection to label respectively the centrioles and microtubules. Centriole mobility was analyzed in living cells by collecting 3D image stacks every 10 min for 4 to 6 hrs. Both fluorescence excitation and differential interference contrast (DIC) were used. DIC images were taken to follow the progression through cell division and used to identify the nuclear envelope and visualize the movement of the cells. As a control for potential transient transfection artifacts, HeLa cells stably expressing centrin1-EGFP [31] were analyzed.

Centrin1-EGFP and α-Tubulin-mCherry were also co-expressed in the p53-deficient mouse mammary tumor cells KP-7.7. Human centrin1 shows only 90% sequence homology to its mouse variant. Correct localization of centrin1 was confirmed by immunostaining for γ-Tubulin in mouse cells expressing the centrin1-EGFP (data not shown).

**Figure 1:**
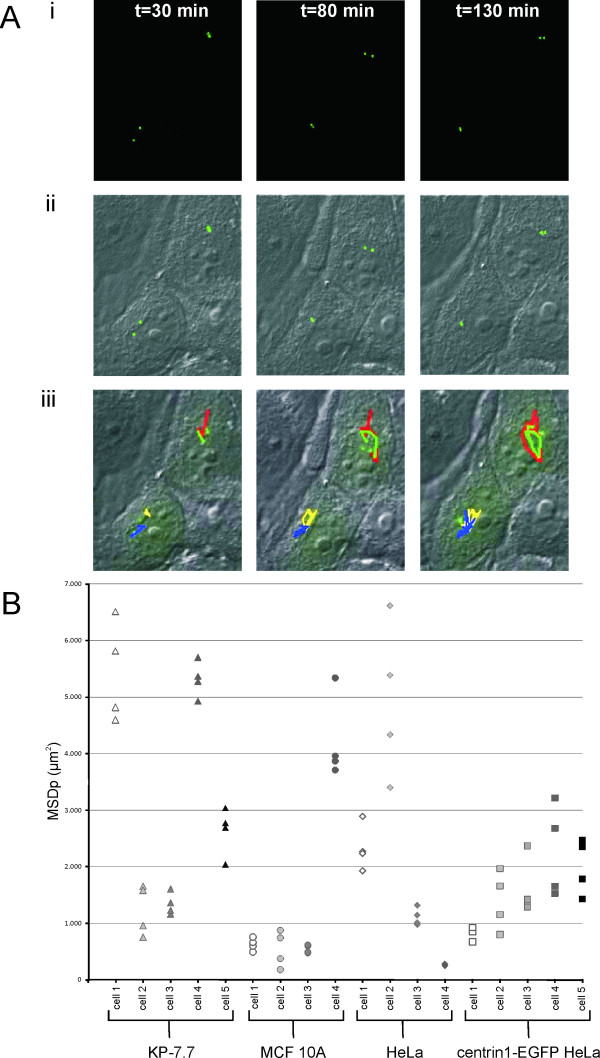
**Tracking centrioles during cytokinesis**. **A) **Representative images of centriole tracks in live MCF 10A cells are shown at three different time points as obtained with the tracking software Stacks (see Additional file 1 movie 1). i) The images show centriole (green) positioning at three different time points, 30 min, 80 min and 130 min after the onset of telophase. ii) The images are an overlay of centrin1-EGFP (green) and DIC. iii) The images are an overlay of centrin1-EGFP (green), DIC and the tracks of the centrioles for 130 min, from the onset of telophase. Every centriole track is represented by a unique pseudo-color and the tracks are determined by linking the centrioles between time-points. Analysis of the tracks revealed that these centrioles moved as fast as 0.35 μm/min on average (see Table 1). **B) **Distribution of the mean square displacement (MSDp) of movements of all 4 centrioles in individual cells (therefore 4 values for every cell) of all cell lines. Different gray tones are used to separate individual cells within a cell line. Triangles represent KP-7.7, circles MCF 10A, diamonds HeLa and squares centrin1-EGFP HeLa. The tracks presented in A) is MCF 10A cell 4. Mobility varied greatly between cells within every cell line. KP-7.7 had the most mobile centrioles (Table 1). MSDp of centrioles was compared using linear mixed model, taking cells as random effects.

By analyzing the movements of centrioles in both human and mouse epithelial cells we noticed that centrioles were highly mobile in all cell lines and moved in different directions with varying speeds. To preclude subjective assessment of centriole mobility, we performed a quantitative analysis of the centriole movements using the tracking software Stacks (Figure 1A and Additional file 1 movie 1). The tracking showed that the mouse epithelial cell line KP-7.7 had the most mobile centrioles from anaphase onset until 60 min after abscission. Their centrioles moved on average 3.2·10^3 ^μm^2^, with the average speed of 0.35 μm/min, which is 1.5-2.7 times faster than measured for centrioles of the human epithelial cell lines tested (Table 1). Cells were taken as random effects when comparing the mean square displacement (MSDp) of centrioles between the different cell lines because of great variations between cells within every sample (Figure 1B). The variance of centriole mobility was calculated to be 10 times greater between cells within a sample than within a cell, 30.3·10^8 ^versus 3.06·10^8 ^respectively.

**Table 1:** Centriole mobility during cytokinesis of various mammalian epithelial cell lines

Cell line	Average MSDp (**μm**^**2**^)	Average speed (μm/min)
**KP 7.7**	3.2·10^3^	0.35
**MCF 10A**	1.5·10^3^	0.13
**HeLa**	2.2·10^3^	0.24
**centrin1-EGFP HeLa**	1.6·10^3^	0.17

Three main types of movements were detected: 1) migration along the nuclear envelope, 2) irregular, where centrioles moved in the cytoplasm or cell center with no specific direction and 3) traveling along microtubules forming the spindle axis.

**Figure 2:**
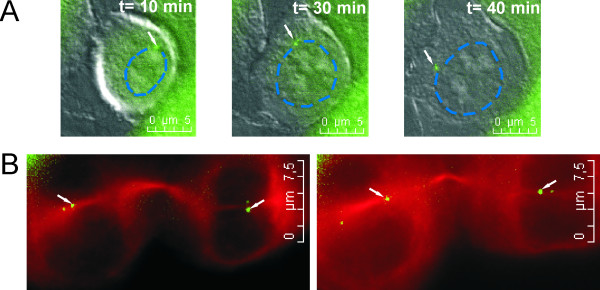
**Centrioles move either along the nuclear envelope or along microtubules containing α-Tubulin**. **A) **Centriole moving along the nuclear envelope in a centrin1-EGFP HeLa cell. The images shown are an overlay of centrin1-EGFP (green) and DIC. An arrow indicates mobile centrioles migrating along the nuclear envelope. The nuclear envelope is emphasized with a blue dotted line. **B) **A dividing HeLa cell showing centrioles that leave the cell pole and migrate through the cell center towards the intercellular bridge along microtubules. White arrows point at centrioles migrating along α-Tubulin towards the intercellular bridge.

Before abscission one of the separated centrioles or the whole centrosome was frequently found to be associated with and to migrate along the newly formed nuclear envelope in G_1 _daughter cells. However, centriole migration was found to vary between daughter cells and between cell lines. A centriole moving along the nuclear envelope was detected in 58% of imaged KP-7.7 cells, 62% of MCF 10A cells, 75% of HeLa cells and 44% of imaged centrin1-EGFP HeLa cells (Figure 2A). Centrioles usually migrated along the nuclear envelope in one direction only, towards the intercellular bridge. Occasionally they detached from the nuclear envelope before reaching the intercellular bridge. Centrioles with a bi-directional movement migrated to the intercellular bridge and then back to the nuclear envelope by the same path. Tubulin foci were detected around the centrosomes at the nuclear envelope where the centrioles showed independent and irregular movements (Additional file 2 Figure S1). This was more frequently seen around immobile centrosomes that stayed close to the cell pole during cytokinesis. When centrioles did not move along the nuclear envelope in the direction of the cleavage site, they migrated through the cell center along microtubules containing α-Tubulin (Figure 2B).

### Migration of centrioles to the intercellular bridge is not essential for abscission in human and mouse epithelial cells

**Figure 3:**
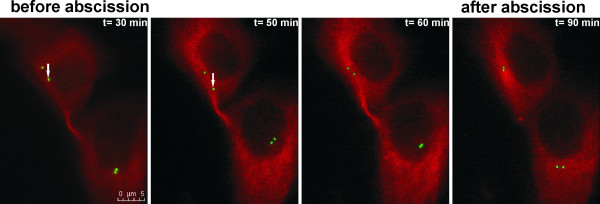
**Centrioles migrate occasionally towards the intercellular bridge during cytokinesis in human epithelial cells**. Representative images of centriole migration to the intercellular bridge prior to abscission (first 3 time points) and after abscission (t = 90 min) are shown for the human MCF 10A epithelial cell line (see Additional file 3 movie 2). The cells were transiently co-transfected with centrin1-EGFP (green) and α-Tubulin-mCherry (red) expression constructs, to label centrioles and the microtubules, respectively. White arrows point at a centriole migrating to the intercellular bridge.

**Figure 4:**
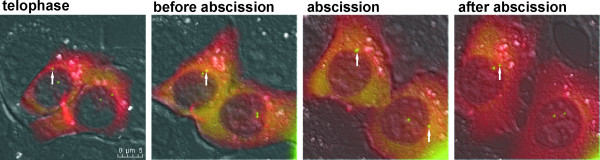
**Centriole movement in mouse mammary epithelial cells during cytokinesis**. During cytokinesis, centrioles show increased mobility and either one centriole or the complete centrosome is frequently associated with the nuclear envelope. After abscission centrioles increase their mobility and frequently detach from the nuclear envelope. The images show an overlay of DIC, green and red recordings to show centriole-specific centrin1-EGFP localization together with the intercellular bridge marker α-Tubulin-mCherry. White arrows indicate centrioles by the nuclear envelope.

In agreement with previously published data [28] we observed separation of mother and daughter centrioles in 90% of dividing cells after the formation of the cleavage furrow. In 3 out of 11 (27%) MCF 10A cells that completed abscission a centriole was observed to migrate to the intercellular bridge (Figure 3, Additional file 3-movie 2). In HeLa cells this was shown to occur 2 times more frequently than in MCF 10A cells (Table 2). No difference in centriole migration was observed between HeLa cells transiently or stably expressing centrin1-EGFP (50% versus 55%). In 7 out of 12 (58%) KP-7.7 cells either a whole centrosome or only the mother or the daughter centriole left the cell pole. This behavior was observed in one or both daughter cells. In mouse cells with mobile centrioles, centrioles showed an irregular movement along the nuclear envelope (Figure 4). Repositioning of a centriole to the intercellular bridge was not observed in any of the KP-7.7 cells (Table 2). Together, these observations suggest that abscission can take place without separation and repositioning of the centrioles.

**Table 2:** Frequency of centriole(s) at the intercellular bridge of various mammalian epithelial cell lines

Cell line	Mobile centriole(s) at the intercellular bridge		Mobile centriole(s) not observed at the intercellular bridge	Immobile centrioles
			
	from one daughter cell	from both daughter cells		
**KP 7.7** (n = 12)	0 (0%)	0 (0%)	7 (58%)	5 (42%)
**MCF 10A** (n = 11)	2 (18%)	1 (9%)	7 (64%)	1 (9%)
**HeLa** (n = 16)	5 (31%)	3 (19%)	3 (19%)	5 (31%)
**centrin1-EGFP HeLa** (n = 22)	10 (46%)	2 (9%)	6 (27%)	4 (18%)

As centriole mobility might be influenced by confluency of the cell culture, the centriole mobility was investigated at different cell densities. Mobile centrioles were more frequently found to reposition to the intercellular bridge when cells were grown at low or intermediate density (up to 70%) as compared with cells grown at high density showing much cell-cell contact, in 35% and 50% of analyzed cells versus 15%, respectively (Additional file 4 Table S1).

When centrioles migrated to the intercellular bridge, the time they remained at or near the bridge before abscission occurred varied between cell lines. In most cells (84%) the centrioles stayed ≤ 30 min at the intercellular bridge, which is in line with previously published data [28]. The main difference between the cell lines was the length of the time interval between the relocation of the centriole from the intercellular bridge until the abscission. MCF 10A cells completed abscission within 30 min, 10 min on average, after the centriole left the intercellular bridge. For HeLa cells, this time period varied from 0 min up to 130 min and was on average 35 min (48 min for transiently transfected HeLa cells and 27 min for stably expressing centrin1-EGFP HeLa cells). Following abscission, the centrioles or even the whole centrosome became more mobile and frequently detached from the nuclear envelope.

We observed no correlation between the migration of a centriole to the intercellular bridge and any special characteristics of microtubule disassembly at the intercellular bridge. We noticed, however, that microtubule particles are released from the midbody and float in the extracellular space (Additional file 5 Figure S2).

## Discussion

In this study we observed that centrioles are highly mobile during cell division and that their movements are different among the mammalian epithelial cell lines studied. During cytokinesis they move primarily along the nuclear envelope and along microtubules containing α-Tubulin. Importantly, repositioning of a centriole to the intercellular bridge was not found to be prerequisite for completion of abscission.

Consistent with previously published data showing that centrosome movement during cytokinesis is microtubule-dependent [26], we observed that in newly formed G_1 _daughter cells centrioles attach to and migrate along the nuclear envelope. In *Caenorhabditis elegans*, centrosomes are tightly associated with the nuclear envelope and dynein, zyg-12 and sun-1 are essential for centrosome attachment to the outer nuclear membrane [32,33]. In line with this, emerin was found to associate with microtubules to link centrosomes to the nuclear envelope [34]. In *Saccharomyces cerevisiae *the spindle pole body (SPB) is anchored in the nuclear envelope by hook-like appendages that originate in the central plaque [35]. The SPB membrane proteins Mps2p and Ndc1 attach the SPB to the nuclear envelope [36,37]. Whether other proteins are involved in the controlled movement of centrioles along the nuclear envelope in mammalian cells remains to be determined.

Abscission is a regulated process. Nuclear and cytoplasmic signaling proteins concentrate on centrosomes and other elements of the mitotic apparatus during G_2_/M transition. After the onset of anaphase, recruitment of myosin II drives the formation of the cleavage furrow in animal cells [17]. Assembly of the contractile ring and ingression of the cleavage furrow to form the intercellular bridge, are key events before abscission. Abnormal furrowing and deficiency of or defects in proteins mediating cytokinesis, as BRCA2, can lead to a delay in the process and more severely, to incomplete cytokinesis [38].

A current model, supported by earlier findings [26], describes that the mother centriole has to reposition from the cell pole to the intercellular bridge and that abscission can take place only when the centriole moves back to the cell center [28]. We observed that centrioles migrate to the intercellular bridge in only about one third of human mammary epithelial MCF 10A cells analyzed and in half of all HeLa cells. In the p53-deficient mouse mammary tumor cells KP-7.7 centrioles were never observed to migrate to the intercellular bridge. It has been stated that cytokinesis rarely fails in various cell lines, including PtK_1_, CV-1, BHK and LLC-PK, in which a centriole does not migrate towards the intercellular bridge [39]. These and our own observations are inconsistent with those published by Piel *et.al *[28]. They studied centriole motility in stable centrin1-EGFP expressing HeLa cells and in two mouse fibroblast cell lines, L929 and 3T3. A centriole was seen to migrate to the intercellular bridge in 90% of the analyzed centrin1-EGFP HeLa cells but in none of the 3T3 cells. We observed centrioles migrating towards the intercellular bridge in 55% of the centrin1-EGFP HeLa cells. The observed differences between those two studies may be the cause of sub-populations within the cell line, which have evolved during sub-culturing and transfers between institutes. Only epithelial cell lines were included in our study, eliminating cell type specific differences. Furthermore, the conditions under which the cells were imaged were kept constant as well as cell density. Notably, in relatively cell dense areas we observed little centriole mobility and at lower cell density we observed centrioles at least twice as frequently by the intercellular bridge.

It is known that cell-cell contact, cell density and cell adhesion can influence centrosome behavior, mitotic progression and ultimately the phenotype of cells. Recent studies demonstrate that the adhesion pattern of cells is conserved. Daughter cells spread precisely as their mother cell and the mitotic spindle is aligned along the traction field which is preserved from progenitor cell to daughter cells. The daughter cells transmit the tension to each other via the intercellular bridge [40]. This suggests that cell adhesion and traction forces are among the key regulators of abscission [28,41,42]. Dubreuil *et.al*. observed that particles were formed at the midbody and released into the extracellular space. They suggested that this might play a role in changing the tension in the intercellular bridge and thereby facilitate abscission [43]. We observed an increase in centriole mobility when daughter cells were beginning to attach to the culture dish (see Figure 2A, 20-30 min). At that time we also noticed that microtubule particles were released from the midbody, which were not necessarily adopted by either of the daughter cells (see Additional file Figure S2).

## Conclusions

In this study we provide evidence that migration of the centriole towards the intercellular bridge is not a key event in regulating abscission. Centrioles are temporarily very mobile during mitosis and show three different types of movements: 1) along the nuclear envelope, 2) irregular, and 3) along microtubules containing α-Tubulin. Based on the observed variation in centriole mobility in different epithelial cell lines we conclude that the movements and positioning of centrioles in late telophase cells, until abscission occurs, is highly cell line dependent.

## Materials and methods

### Cell lines

HeLa cells were cultured in Dulbecco's Modified Eagle's Medium (DMEM) supplemented with 10% fetal bovine serum (FBS), 100 units/ml penicillin and 0.1 mg/ml streptomycin (all from Invitrogen Corporation, Breda, The Netherlands). HeLa cells stably expressing centrin1-EGFP (kindly provided by Dr. Fanni Gergely, Cancer Research UK Cambridge Research Institute, Cambridge, England, with permission from Dr. Matthieu Piel [31]) were cultured using the same culture medium supplemented with 0.4 mg/ml Geneticin (Invitrogen Corporation). MCF 10A (American Type Culture Collection) cells were cultured in DMEM/F12 supplemented with 5% horse serum, 100 units/ml penicillin, 0.1 mg/ml streptomycin, 20 ng/ml epidermal growth factor (EGF) (from Invitrogen Corporation), 10 μg/ml insulin, and 0.5 μg/ml hydrocortisone (both from Sigma-Aldrich Chemie B.V., Zwijndrecht, The Netherlands) and 0.1 μg/ml cholera toxin (Gentaur, Kampenhout, Belgium). The KP-7.7 cell line (kindly provided by Dr. Jos Jonkers, The Netherlands Cancer Institute, Amsterdam, The Netherlands) was derived from a p53-deficient mouse mammary tumor as described [44] and cultured in DMEM/F12 (Invitrogen Corporation) supplemented with 10% FBS, 100 units/ml penicillin, 0.1 mg/ml streptomycin, 5 ng/ml EGF, 50 μg/ml insulin and 5 ng/ml cholera toxin. All cells were cultured in a 5% CO_2 _humidified incubator at 37°C.

### Transfection

Cells were transiently transfected at approximately 30% confluency with centrin1-EGFP and α-Tubulin-mCherry expression constructs by using Lipofectamine™ 2000 according to manufacturer's instructions (Invitrogen Corporation). Centrin1-EGFP [31] was kindly provided by Dr. Michel Bornens, Institute Curie, Paris, France and YFP-α Tubulin [45] by Dr. Jan Ellenberg, EMBL, Heidelberg, Germany. YFP was exchanged for mCherry by performing a polymerase chain reaction (PCR) on a mCherry expression construct [46] (kind gift of Dr. R. Tsien, Howard Hughes Medical Institute, University of California, San Diego, USA), with the following forward and reverse primers; 5' ATATAGCTAGCGCTACCGGTCGCCACCATGGTGAGCAAGGGCGAGGAG 3', 5' TATATCTCGAGATCTGAGTCCGGACTTGTACTTGTACAGCTCGTCCATGCC 3', respectively. After gel extraction, purification of the PCR products and digestion with *Xho*I and *Nhe*I restriction enzymes (New England BioLabs, Westburg B.V., Leusden, The Netherlands) at 37°C for 2 hrs subsequent purification steps were performed according to protocol. YFP-α-Tubulin construct was digested with the same restriction enzymes and after gel extraction and purification the mCherry sequence was ligated to the α-Tubulin expression construct.

### Time-lapse live-cell imaging

Transiently transfected cells cultured in glass-bottom culture dishes (MatTek) were imaged at approximately 70% confluency 48 hrs after transfection. Cytokinesis and centriole movements were recorded with a Leica AF6000 LX microscope system equipped with an inverted DMI 6000B microscope, a DFC350 FX monochrome digital camera (1.4 Megapixel, 12 bit) and a climate chamber (Leica Microsystems). 3D image stacks were collected, each stack containing about 10 optical sections with 1 μm thickness, every 10 min for up to 6 hrs using a HCX PL APO 63×/1.30 GLYC [ne = 1.460 CORR 37°C] objective embedded in glycerin solution (Leica Microsystems). Cells were exposed to both differential interference contrast (DIC, also known as Nomarski microscopy) transmitted light as well as fluorescence light. A TexasRed filter was used to visualize mCherry and a B/G/R filter with separate FITC excitation to visualize green fluorescence emission. The LAS AF software (Leica Microsystems) was used to process the collected time-lapse images and to generate movies. Temperature, CO_2 _concentration and humidity were strictly controlled and kept constant to avoid induction of stress responses, which could influence the processes under study [47]. In addition, the size of image stacks, exposure times and the number of exposures were kept to minimum in all experiments as those factors are known to induce free radical formation and photo damage [48,49].

### Tracking of centriole movements

An in-house developed tracking software, Stacks, was used to quantitatively analyze centriole kinetics. This program allows visualization of time-lapse 2D and 3D image data, offers movie facilities, and provides great flexibility to enhance, process and analyze image stacks. To track the centrioles, image segmentation was performed using global thresholding and additionally the threshold was adjusted for each slice in a time series or in case of one slice at a specific time-point. Following segmentation, the position, size and total density of each centriole particle was measured for all time-points. Then tracks were determined by linking the centrioles between successive time-points, which had the highest calculated probability based on these features. Every identified centriole was assigned with a unique pseudo-color, so that we could identify which centriole was classified as being the same when scrolling between time-points. By manual, interaction tracks were split and reconnected to correct for errors made by the automatic procedure. Finally kinetic parameters such as mean squared displacement (MSDp) were calculated to characterize the mobility of individual centrioles.

### Statistical analysis

The null hypothesis stating that all cells tested would reveal an equal mean value of the mean square displacement was tested by using linear mixed model calculations. Cells were taken as random effects as there were great variations of centriole mobility between cells within every cell line.

## Authors' contributions

ABJ participated in the design of the study, setup and performed the experiments, processed the data, participated in interpretation of the results and drafted the manuscript. RWD, HMÖ, HJT, JEE and KS participated in the design of the study, assisted with interpretation of the data and drafting the manuscript. RWD, HMÖ and KS critically revised the manuscript. JV programmed the Stacks computer software, assisted with processing the data and with calculations. HJT, JEE and KS supervised the project. All authors read and approved the final manuscript.

## Supplementary Material

Additional file 1 **Centriole tracks in MCF 10A cells during cytokinesis**. Centrioles were tracked using the tracking software Stacks (see Figure 1A). Every centriole track is represented by a different color. Click here for file

Additional file 2 **Centrosome is mobile in an α-Tubulin net**. Centrosome show low mobility in α-Tubulin foci close by the nuclear envelope (inset). Blue-dotted lines represent the nuclear envelope. Images shown are an overlay of centrin1-EGFP (green) and α-Tubulin-mCherry (red). Click here for file

Additional file 3 **Centriole mobility in MCF 10A cell during cytokinesis**. Representative movie of centriole mobility in epithelial MCF 10A cells during cytokinesis (see Figure 3). Images were collected every 10 minutes using fluorescent excitation and DIC. The centrosome compartments centrioles are marked green and in red are microtubules. Microtubules accumulate and form the intercellular bridge during cytokinesis. A centriole migrates to the intercellular bridge before abscission occurs. Click here for file

Additional file 4 Frequency of centriole repositioning to the intercellular bridge in relation to cell density. Click here for file

Additional file 5 **Midbody in extracellular space after abscission**. Representative images of release of microtubule particles from the intercellular bridge and fate of midbody after abscission. Intercellular bridge containing the midbody (white arrows) links the two daughter cells. During abscission the bridge is cut. The midbody floats in the extracellular space after abscission. Click here for file
