# Supplementary figures and images for: Centriole movements in mammalian epithelial cells during cytokinesis
Source: BMC Cell Biol. 2010 May 21;11:34. doi: 10.1186/1471-2121-11-34 (PMC2893098; doi:10.1186/1471-2121-11-34)

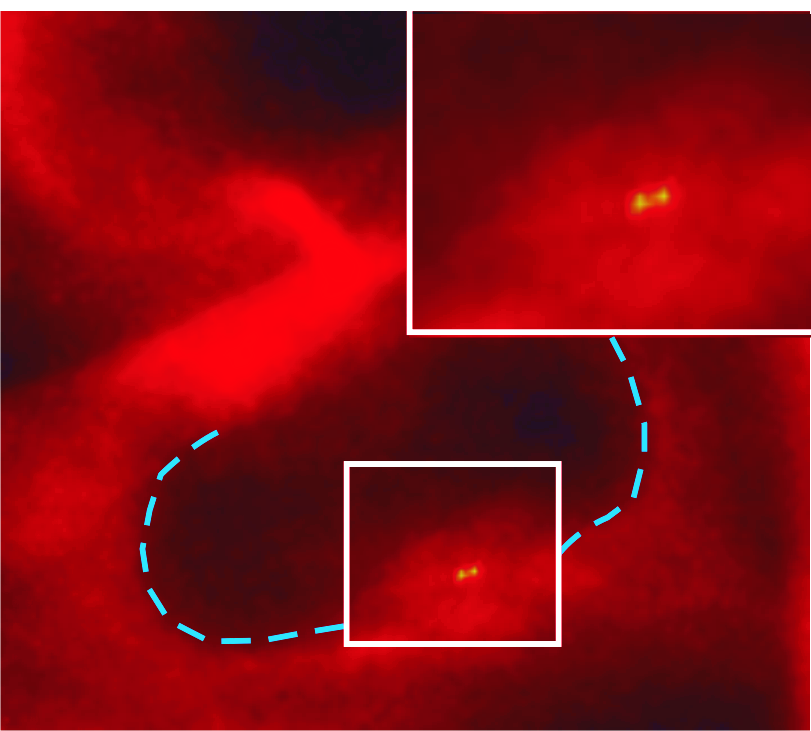

Supplement: Additional file 2 — Centrosome is mobile in an α-Tubulin net. Centrosome show low mobility in α-Tubulin foci close by the nuclear envelope (inset). Blue-dotted lines represent the nuclear envelope. Images shown are an overlay of centrin1-EGFP (green) and α-Tubulin-mCherry (red). [file 1471-2121-11-34-S2.TIFF]

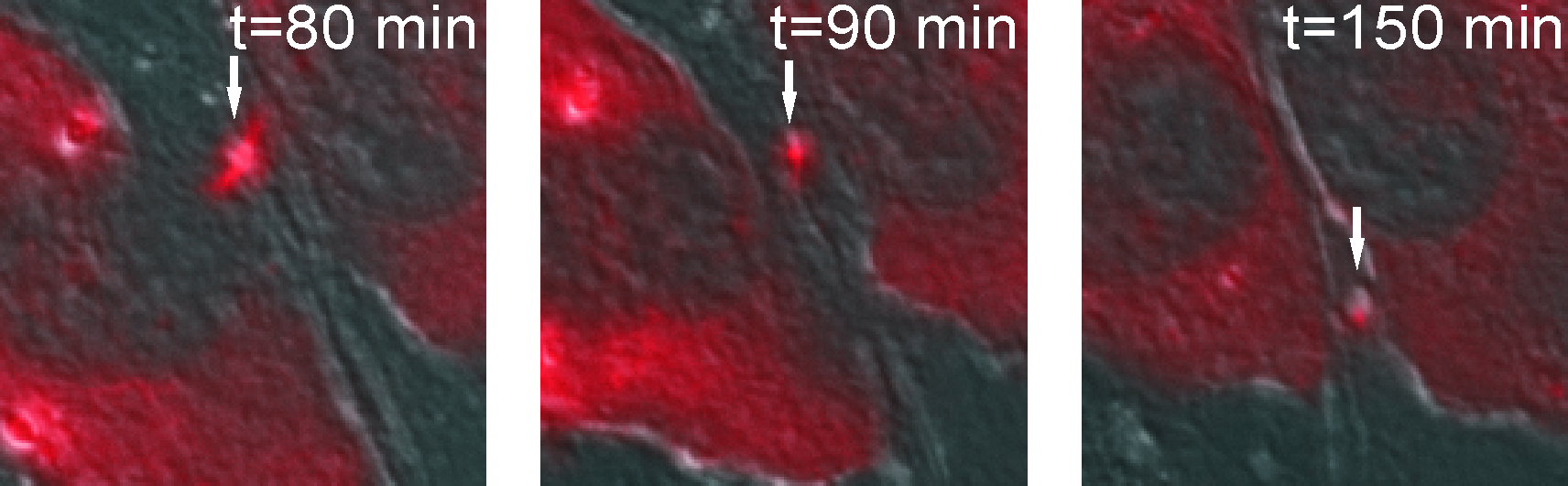

Supplement: Additional file 5 — Midbody in extracellular space after abscission. Representative images of release of microtubule particles from the intercellular bridge and fate of midbody after abscission. Intercellular bridge containing the midbody (white arrows) links the two daughter cells. During abscission the bridge is cut. The midbody floats in the extracellular space after abscission. [file 1471-2121-11-34-S5.TIFF]
