# Supplementary material for: Centriole movements in mammalian epithelial cells during cytokinesis
Source: BMC Cell Biol. 2010 May 21;11:34. doi: 10.1186/1471-2121-11-34 (PMC2893098; doi:10.1186/1471-2121-11-34)
Supplement: Additional file 4 — Frequency of centriole repositioning to the intercellular bridge in relation to cell density. [file 1471-2121-11-34-S4.PDF]

**Additional file 4 Table S1 – Frequency of centriole repositioning to the intercellular bridge in relation to cell density**

| Mobility | Low cell density (%) | Normal cell density (%) | High cell density (%) | **Total** |
|---|---|---|---|---|
| **Mobile centriole(s) at the intercellular bridge** | 7 (35%) | 10 (50%) | 3 (15%) | 20 (100%) |
